# Molecular characterization of China rabies virus vaccine strain

**DOI:** 10.1186/1743-422X-8-521

**Published:** 2011-11-17

**Authors:** Wenqiang Jiao, Xiangping Yin, Zhiyong Li, Xi Lan, Xuerui Li, Xiaoting Tian, Baoyu Li, Bin Yang, Yun Zhang, Jixing Liu

**Affiliations:** 1 State Key Laboratory of Veterinary Etiological biology, Key Laboratory of Epizootic disease of Grazing Animal of Ministry of Agriculture, Lanzhou Veterinary Research Institute, Chinese Academy of Agricultural Science(LVRI, CAAS), Xujia ping1, Yanchang bu, Lanzhou, Gansu, Post Code 730046, China

**Keywords:** Rabies virus, aG, China, full-length genome

## Abstract

**Background:**

Rabies virus (RV), the agent of rabies, can cause a severe encephalomyelitis in several species of mammals, including humans. As a human rabies vaccine strain employed in China, the genetic knowledge of the aG strain has not been fully studied. The main goal of the present study is to amplify the whole genome of aG strain, and genetic relationships between other vaccine strains and wild strains were analyzed.

**Results:**

The entire genome of human rabies virus vaccine strain aG employed in China was sequenced; this is the second rabies virus vaccine strain from China to be fully characterized. The overall organization and the length of the genome were similar to that of other lyssaviruses. The length of aG strain was 11925nt, comprising a leader sequence of 58nt, nucleoprotein (N) gene of 1353nt, phosphoprotein (P) gene of 894 nt, matrix protein (M) gene of 609nt, glycoprotein (G) gene of 1575nt, RNA-dependent RNA polymerase (RdRp,L) gene of 6384nt, and a trailer region of 70 nt. There was TGAAAAAAA (TGA_7_) consensus sequence in the end of each gene, except AGA_7 _at the end of G gene. There was AACAYYYCT consensus start signal at the beginning of each gene.

**Conclusions:**

In this report, we analyzed the full genome of China human rabies vaccine strain aG. Our studies indicated that the genome of aG retained the basic characteristics of RV. At gene level, N was the most conserved among the five coding genes, indicating this gene is the most appropriate for quantitative genotype definition. The phylogenetic analysis of the N indicated the aG strain clustered most closely with Japanese and Russian rabies vaccine strains, suggesting that they may share the same ancestor; also, the aG strain did not share high homology with wild strains isolated from China, making it may not be the best vaccine strain, more research is needed to elucidate the genetic relationship among the RV circulating in China.

## 1. Background

Rabies is a widespread neurological zoometric disease which affects almost all kinds of mammals, including humans. The morality is almost 100%. It is reported that nearly 55,000 human die of rabies all over the world each year, most occurred in Asia and Africa [1]. Following India, China has the second highest number of human cases in the world [2]. The causal agent of the disease is rabies virus (RV), a member of Lyssavirus genus, Rhabdoviridae family. The RV genome is almost 12 kb in size consisting of five monocistronic RNAs, which encodes the nucleocapsid protein (N), phosphoprotein (P), matrix protein (M), glycoprotein (G) and RNA-dependent RNA polymerase (large protein). Between the monocistronic there are four intergenic regions (IGRS) with different length [3]. Besides, at the end of the genome there are two underinflated regions (UTRS), which play an important role in virus replication.

So far, vaccination is the most effective method to prevent rabies. The vaccine strains employed in China are CTN, aG, PM and PV. The complete genome sequence of CTN strain has been sequenced already [4]. Although novel vaccines, such as oral vaccine and live attenuated vaccine have been developed in recent years, the virus strain used for the production of vaccine are still pathogenic for laboratory and wildlife spices, what is more important, these rabies vaccines have the potential of causing vaccine-induced rabies and appear to have limited efficacy in a broad spectrum of animal species [5]. Therefore, genetic knowledge of vaccine strain is essential. Also, comparison of sequence of vaccine strain and field strain circulating in the country would prove how effective of the vaccine against the field virus [6].

The aG strain, also known as pG strain, was obtained from a rabid dog in 1931, Beijing. After 50 passages in the brain of rabbit, the strain was named aG strain. Then, the aG strain has been adapted to VERO cell cultures after 68 passages. Much higher antibody was obtained in immunized dogs, and the morality was lower. Since then, the aG strain has been chosen for the production of human rabies vaccine. Before this study, P, M, G and L genes of aG strain have been sequenced; however, this is the first time that the complete genome has been sequenced. In the present paper, the complete sequence of the rabies virus vaccine strain aG employed in China was sequenced. The sequence obtained indicates that aG strain genome is similar to the sequence of other complete lyssavirus genome available in Genbank. Sequence comparison with other rabies virus strain and phylogenetic analysis according to the N gene also falls within the scope of this study.

## 2. Materials and methods

### 2.1. Virus strain

The aG strain was prepared and stored by State Key Laboratory of Veterinary Etiological Biology, Lanzhou Veterinary Research Institite, Chinese Academy of Agricultural Science (LVRI, CAAS). The parental virus was isolated from a rabid dog's brain from Beijing, China, of a patient died of rabies from Beijing 1931, China. After 31 passages in primary hamster kidney (PHKC) cell, it was chosen for vaccine production in China [4].

### 2.2 Primer design

According to the conserved regions of the genome of rabies virus published in GenBank, particular the RC-HL(Genbank number: AB009663), RV-97(Genbank number EF542830), Nishigahara(Genbank number: AB044824) and NI-CE(Genbank number: Ab128149), 12 pairs of primers were designed to amplify the unknown regions of aG strain by primer premier 5.0 software(Table 1). All primers were synthesized by Shanghai Sangon Biological Engineering Technology & Service Co., Ltd.

**Table 1:** Primers employed for the amplication of unknown genomic RNA of rabies virus aG strain

Fragments	Sequence(5'-3')	Lengths(bases)	Base position
F1^a^F^b^	ACGCTTAACAACAAGATCA	19	1-1581
F1R	CTTCAGCCATCTCAAGAT	18	
F2F	TATGTCTCAGTCAGTTCC	18	1340-2933
F2R	TCATCCCAAGTGATCTCC	18	
F3F	ATACTCTGGGAATCATAGGAT	21	2742-4280
F3R	TGAGACGTCTGAAACTCACTG	21	
F4F	AACATGGGTCGCGATGCA	18	4090-5453
F4R	GGTCATCATAGACCTCTC	18	
F5F	CATGTAGATTCTCATAAT	18	5063-6410
F5R	TAAATACAGGAAAGTCTC	18	
F6F	TGTGGAAACTCCGGCTAT	18	6290-7430
F6R	CGATGAGGTCTGATCTGTCTG	21	
F7F	TGTTTGGATTGAAGAGAGTGT	21	7344-8513
F7R	GCTCACTGAGAAATCGAG	18	
F8F	TTCAGAGTTTCGAGAGGCAAT	21	8410-9648
F8R	ATGTTGACAGGGAAGATGGTT	21	
F9F	ATGTTCCAGCCATTGATGCTT	21	9272-10598
F9R	TGAACACAAGCTTGGCATCAG	21	
F10F	TGCTCTGCTCAACAGGTT	18	10331-11932
F10R	ACGCTTAACAAATAAACAACA	21	
F11F	GGTCTGGCGACACCCCGGTGTTCA	24	
F11R	GAGTCTGTCATCTCACTGGATCA	23	
F12F	TGCTGCTGCCAAGTAGGAACAT	22	
F12R	AGTTTGGCGGCATCCATGCCTG	22	

a The 12 fragments are labeled from "F1" to "F12"

b "F" and "R" strand for forward and reverse direction of the primer extension

### 2.3 Reverse transcription-PCR and direct sequencing

Total RNA were extracted from lyophilized brains of mice infected with aG strain by using an Rneasy Mini Plus Total RNA extraction kit (Qiagen) according to the manufacture's instruction. Single cDNA was synthesized by using First Strand cDNA Synthesis Kit (ToYOBO), and the cDNA were amplified using KOD Plus Neo (TOYOBO). Amplified DNA products were examined via electrophoresis using 0.8% ultrapure TM Agarose (Invitrogen) gels. DNA products of expected size were purified using Agarose Gel DNA Purification Kit (TAKARA). The purified products were added to A at the end of 3'end using DNA A-tailing Kit (TAKARA) and then ligated with pMD18-T (TAKARA), the ligated products were transformed into competent cell JM109 (TianGen), at least 3 clones carrying the PCR products of expected size were identified by PCR and the positive clones were sequenced commercially (Shanghai Sangon biological Engineering Technology &Services Co., Ltd., China).

### 2.4 Amplification and sequencing of the terminal ends

The 5'and 3'terminal of the genome was confirmed using the approach described by Kuzmin et al [7]. Briefly, Total RNA was subjected to ligation by T4 RNA ligase (Invitrogen) to circularize the genome. Thereafter the ligated RNA was subjected to nested PCR with the primer marked with F 11F, 11R, 12F and 12 R located within the 5'and 3'end of the genome. Products of the expected size were dealt with above.

### 2.4 Sequence alignment and analysis

Nucleotide sequences obtained were edited and assembled manually using EditSeq of DNAStar program 7.0 (DNAStar). Phylogenetic analysis of N gene of Lyssavirus, including the construction of phylogenetic tree were conducted using Molecular Evolutionary Genetics Analysis (MEGA) Version 5.0 with the methods of neighbor-joining (NJ) algorithm with the Kimura two-parameter model. The reference sequences have been listed in Table 2. The reliability of the phylogeny groupings was evaluated using bootstrapping with 1000 replicates. Bootstrap values of 70% or greater were viewed significant.

**Table 2:** Complete genome of rabies virus used in this study from Genbank

Strain	Origins	Country	Accession no	Length
CTN181	Attenuated vaccine strain, derived from the brain tissue of a patient in Shandong, China	China	EF564174	11923 bp
Nishigahara	Seed strain of animal rabies vaccine strain used in Japan	Japan	AB044824	11926 bp
SAD-B19	A highly attenuated mutant of SAD strain	America	AB044824	11928 bp
RC-HL	Animal vaccine strain used in Japan	Japan	AB009663	11926 bp
RV-97	Vaccine strain employed in Russia	Russia	EF542830	11932 bp
PV	Pasteur vaccine strain	France	M13215	11932 bp
ERA	Attenuated rabies vaccine strain	America	EF206707	11931 bp
SRV9	Avirulent vaccine strain	China	AF499686	11928 bp
NI-CE	Developed from Nishigahara	Japan	AB128149	11926 bp
HEP-Flury	Derived from a human isolate in Georgia, USA, 1939	America	AB085828	11615 bp
DRV	Isolated from a deer in Ji lin, China	China	DQ875051.	11863 bp
MRV	Isolated from a mouse in China	China	DQ875050.	11869 bp
HN10	Isolated fron a rabies patient in Hunan, China	China	EU643590	11923 bp
SHBRV-18	Isolated from a silver-haired bat	America	AY705272	11923 bp
BD06	Isolated in China	China	EU643590	11924 bp
NNV-RAB-H	Isolated from the brain of human	India	EF437215	11928 bp
SAG2	Vaccine strain Derived from SAD	America	EF206719	11928 bp
8743THA	A street strain	Thailand	EU293121	11923 bp
8764THA	A street strain	Thailand	EU293111	11925 bp
H-08-1320	Isolated from the brain of human	Sri Lanka	AB569299	11926 bp
RRV ON-99-2	Isolated from a raccoon	Canada	EU311738	11923 bp
BR-Pfx1	Isolated from Dusicyon sp	Brazil	AB362483	11924 bp
9704ARG	Isolated from tadarida brasiliensis	Argentina	EU293116	11923 bp
9147FRA	Isolated from fox	France	EU293115	11923 bp
9001FRA	Isolated from dog	Guyana	EU293113	11922 bp
FJ08	Isolated from dog	China	FJ866835	11924 bp
FJ09	Isolated from dog	China	FJ866836	11924 bp
D01	Isolated from the brain of dog	China	FJ12193	11925 bp
D02	Isolated form the brain of dog	China	FJ12194	11925 bp
MOKOLA	Isolated from Mokola Virus	Africa	NC_006429	11940 bp
KRC5-04	Isolated from dog	South Korea	AY730597	1353 bp
HNDB28	Isolated from dog	China	EU008922	1353 bp
HNDB12	Isolated from dog	China	EU008920	1353 bp
GXPL	Isolated from Canis lupus familiaris	China	GQ472474	1353 bp
Hebei0(H)	Isolated from homo sapiens	China	EU267777	1353 bp
Zhejiang Wz1(H)	Isolated from homo sapiens	China	EU700032	1353 bp
West Caucasian bat virus	Isolated from West Caucasian bat	Russia	EF614258	12278 bp
Flury-LEP	Derived from a human isolate in Georgia, USA, 1939	America	DQ099524	11711 bp
JS08-45	Isolated from Chinese ferret badger	China	GU647092	11922 bp

## 3. Results

### 3.1 Genome organization of the aG strain

Using a total of 24 primers (as shown in table 1) the strain aG genome was obtained as 12 separate overlapping PCR products. The 5'- and 3'-terminal were obtained as mentioned above. The length of the genome is 11925 nucleotides(nt), and the genome organization of aG strain, which follows typical rabies virus organization, is summarized as follows: a 3' leader region of 58 nt(1-58), the N gene (59-1482), P gene (1485-2476), M gene (2482-3284), G gene (3290-5356), L gene (5381-11855), and the 5'trailer region of 70 nt (11855-11925). The coding sequence (CDS) of each of the five structural genes are as follows:1353-nt nucleotide (71-1423), 894-nt phosphoprotein(1515-2408), 609-nt matrix protein (2497-3105), 1575-nt glycoprotein (3317-4891) and 6384-nt large protein(5411-11794). The G-L non-coding region is 455nt. The sequences of the transcription initiation sites (TIS) and the termination signals of the structural genes and the intergenic regions (IGRS) among these genes are shown in table 3.

**Table 3:** Transcription initiation and termination signals for all strain aG

Gene or region	Termination signal	IGRs	Initiation signal
3'leader/N	------	-------	AACACCTCT
N/P	TGAAAAAAA	CT	AACACCCCT
P/M	TGAAAAAAA	CGAGC	AACACCACT
M/G	TGAAAAAAA	CTATT	AACATCCCT
G/L	AGAAAAAAA	CTGTAGATCGAAAGAGCAACTGGC	AACACCTCT
L/5'trail	CGAAAAAAA	-------------------------------------------------	-------

### 3.1.1. Nucleotide feathers of strain aG

The length and sequence of leader region is highly conserved in all lyssavirus. The length of the leader region is 58nt. The first 12 nucleotides, especially the first 9 are the same in all the lyssavirus. However, the conservation is not so stringent after residue 25. Following most lyssavirus, the trailer regions of aG strain is 70nts.

**Figure 1:**
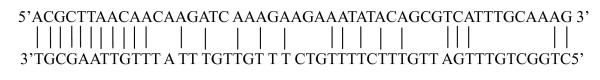
**Comparison of the 3' and 5' genomic termini of the antigenomic (+) sense RNA of aG strain**.

The complementarity of the 3'and 5' ends of the genome is another typical feather of the Mononegavirals, with up to 16 nts are complemented. The first 9 nts of the genome leader regions are absolutely complementary to the corresponding regions of the trailer regions [7] (Figure 1).

The transcription initiation signals (TISs) and transcription termination signals (TTS) are conserved as AACAYYHCT and G (A) _7_, but the TTS between G-L was AGAAAAAAA at the N-P junction. The intergenic regions between the cistrons CT, CCGAA, ACTATT and CTGTAGATCGAAAGAGCAACTGGC (Table 3). Pairwise comparisons of nucleotide and predicted amino acid sequence identities of the aG with the vaccine strain of lyssaviruses are listed in Table 4.

**Table 4:** Pairwise comparisons of nucleotide and predicted amino acid sequence identities of the aG with the vaccine strain of lyssaviruses

Strain		N (%)	P (%)	M (%)	G (%)	L (%)
SRV9	Nucleotide	92.5	89.6	91.1	91	92.2
	Amino acid	96.2	88.9	87.7	89	95.3
CTN181	Nucleotide	87.3	82.9	83.9	84.1	25.1
	Amino acid	96	87.2	93.1	88.8	5.9
ERA	Nucleotide	92.6	89.7	92.3	91.4	92.4
	Amino acid	96.2	89.3	90.6	89.9	95.9
Flury-LEP	Nucleotide	92.1	88.6	92.6	90.5	91
	Amino acid	96.9	87.9	93.6	91.2	96.5
Flury-HEP	Nucleotide	92.1	87.9	91.5	89.8	91.5
	Amino acid	96.9	87.2	92.1	89.9	96.4
NI-CE	Nucleotide	97.9	95.6	96.9	97.1	98.6
	Amino acid	98.4	93.3	96.1	96.6	98.6
Nishigahara	Nucleotide	98.1	96.8	97.2	97.1	98.2
	Amino acid	99.1	95	97	96.4	98.7
PV	Nucleotide	92.3	89.6	92	91.6	92.3
	Amino acid	95.8	89.9	90.6	90.5	95.5
RV97	Nucleotide	95.9	92.5	92.3	93	94.9
	Amino acid	96	87.9	90.6	89.7	96.1
SAD-B19	Nucleotide	92.5	89.6	91.1	91.2	92.2
	Amino acid	96.2	88.9	87.7	89.7	95.3
SHBRV-18	Nucleotide	84.2	79.6	80.8	80	82
	Amino acid	94.7	83.9	88.7	84.2	5.8

### 3.2 Structural feathers of the proteins of aG strain

**Figure 2:**
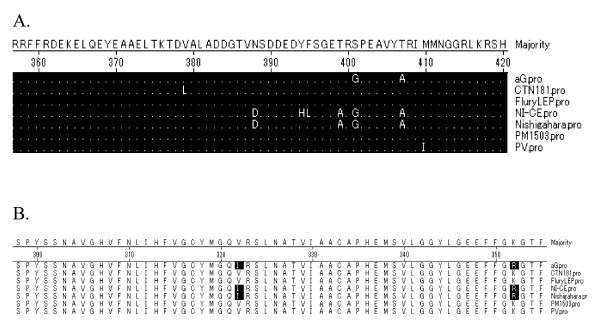
**Alignment of Antigenic sites I and IV, RNA-binding site of N**. Dots represent identity to the majority, shade (with solid black) residues differ from consensus. A. Alignment of Antigenic sites I and IV. Dots represent identity to the majority. B. Alignment of RNA-binding site of N, shade (with solid black) residues differ from consensus.

Consistent with previous studies, at the nucleotide level N was the most conserved gene [8]. The N protein of aG strain is 450 amino acid residues. Only 10_Ala _amino acid substitution is unique to the strain aG. Antigenic site I (residues 358-367), antigenic site IV (375-383, 359-366) and RNA-binding domain (residues 298-352) were found to be conserved in all the isolates analyzed. Ser389, which was considered to be related to casein-type phosphorylation site and regulation of viral RNA transcription and replication, was highly conserved [9] (Figure 2).

**Figure 3:**
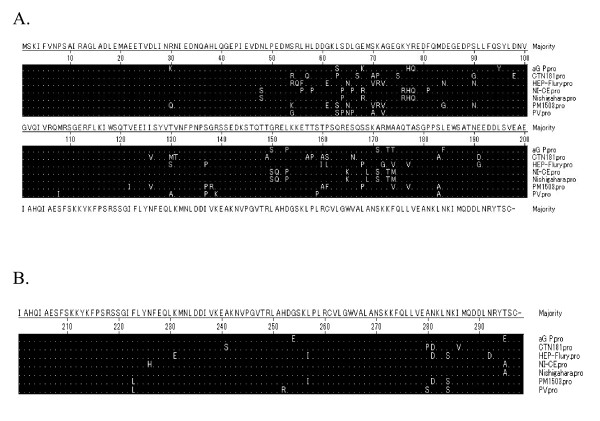
**Alignment of N binding sites (69-177), (268-297)and [(K/R) XTQT], dots represent identity to consensus**. A. Alignment of N binding sites (69-177) and [(K/R) XTQT], dots represent identity to consensus. B. Alignment of N binding site (268-297), dots represent identity to consensus.

P gene was considered to be the most divergent protein among the five structural proteins. Six amino acid substitutions, Lys_68_, Tyr_95_, Thr_174_, Thr_184_, Glu_254 _and Glu_295_, unique to the aG were observed. The interaction of the motif [(K/R)XTQT] between residues 145-149 with cytoplasmic dynein light chain (LC8), which plays an important role in viral nucleocapsid axoplasmic transport, was encoded as KSTQT [10]. Compared with vaccine strain, the N protein binding site (69-177 and 268-297) of aG strain had one unique amino acid residues substitution, Tyr_95_. The L binding site in P, the first 19 amino acid residues, was absolutely conserved in aG. Ser_162_, Ser_210_, Ser_271_, which have been shown to be involved in the phosphorylation of P were well conserved in the aG strain [11] (Figure 3).

**Figure 4:**
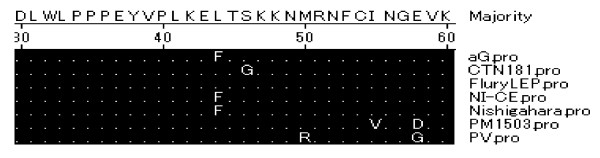
**Alignment of proline-rich motif and residue 58. Dots represent identity to the consensus**.

M protein connects the plasma membranes, RNPS and G protein together, and is deeply involved in the budding of rabies virus. Thr_100_, Ser_111 _and Ser_174 _were the three amino acid substitution to the strain aG. The motif proline-rich (PPxY) between residues 35-38 is thought to be associated with the interaction with WW domains of cellular components, and was encoded PPEY, which is very conserved [12]. Residue 58, which is crucial for regulation of RV RNA synthesis [13] (Figure 4), was encoded Glu in aG strain and Gly in PV strain.

**Figure 5:**
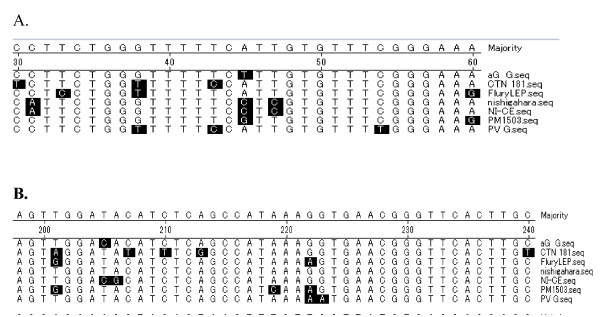
**Alignment of antigenic antigenic siteI (residue 231), site II (residues 34-42), (residues 198-200), shade (with solid black) residues differ from the consensus**. A. Alignment of antigenic site II (residues 34-42), shade (with solid black) residues differ from the consensus. B. Alignment of antigenic site I (residue 231) and II (residues 198-200), shade (with solid black) residues differ from consensus.

The G protein of 514 amino aicd residues is the only viral protein exposed on the surface of the virus and responsible for the immune responses of the host. Compared with vaccine strain analysed in this study, there are eight amino acid substitutions unique to aG strain: His_69_, Pro_184_, Pro_250_, Gly_427_, Ile_431_, Ile_477_, Lys_481_, Asn_160_. Antigenic site I (residue 231), antigenic site II(residues 34-42,198-200), antigenic site III (residues 330-338), antigenic site IV(residue 264) and antigenic site a (residue 342) were conserved. Ala_242_, sp_255_, Ile_268 _and Arg_333_, which are considered to be associated with viral pathogenicity, were replaced with Cys, Met, Thr and Ala respectly (Figure 5).

L protein is an important component of RNP, although the N and P protein are also needed to form RNP. The 2,127 amino acid residues of L protein are distributed into six conserved motifs.

**Figure 6:**
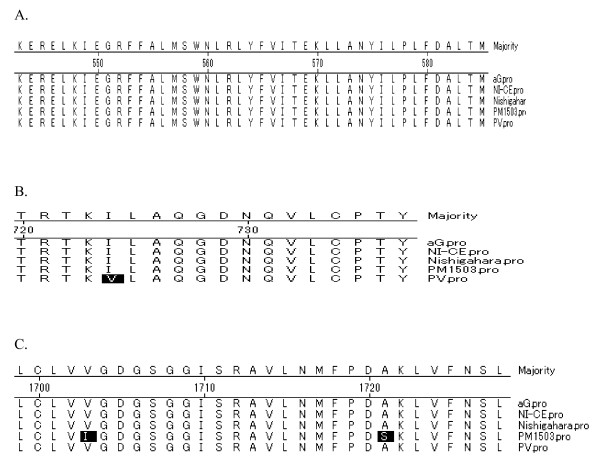
**Alignment of motif A, B, C**. shade (with solid black) residues differ from consensus. A Alignment of motif A. shade (with solid black) residues differ from consensus. B Alignment motif B. shade (with solid black) residues differ from consensus. C. Alignment of motif C. shade (with solid black) residues differ from consensus.

Three potential N-glycosylation sites, which are located at 37-39, 158-160 and 319-321 were conserved in aG. The L gene of aG encoded a protein of 2,127-amino acid, and thirteen amino acid substitutions unique to strain aG were observed: Gly_48_, Gly_58_, Gln_189_, Tyr_353_, Gly_489_, Gly_496_, Ser_606_, Lys_995_, Val_1043_, Ser_1133_, Tyr_1588_, Leu_1658_, His_1801_. The motifs among 544-563(A), 728-732(B) and 1705-1710(C) have been regarded as functional motifs. These motifs are involved in RNA binding, active site of polymerase, polyadenylation or a core of ATP binding site. All these amino acid residues were conserved in strain aG (Figure 6). In the absence of clear functions for these residues, the importance of any of these changes is presently unknown.

### 3.2 Phylogenetic analysis of N

**Figure 7:**
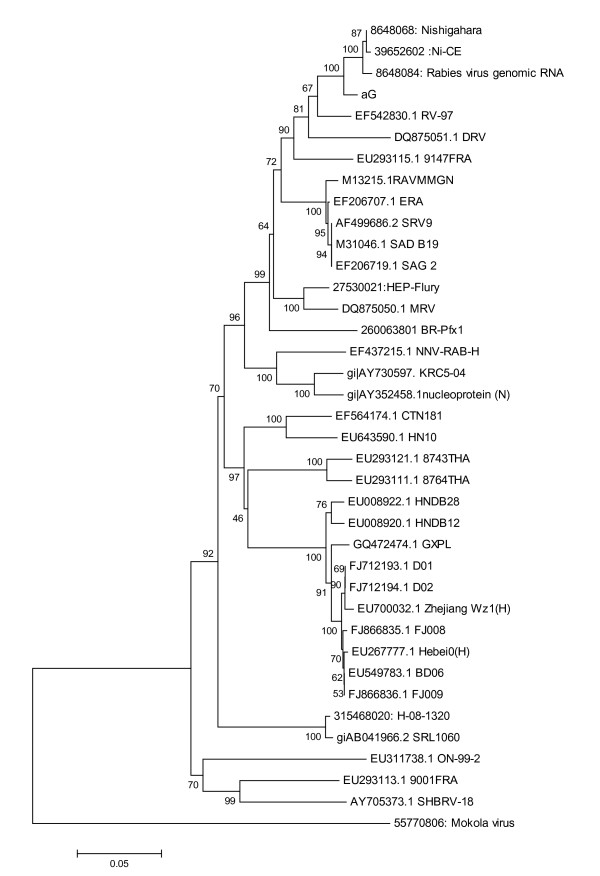
**Phylogenetic analysis of N gene sequence of aG with nucleoprotein sequence available in Genbank by Neighbor-Joining program in MEGA 5 software**. The number below the brace is bootstrap values for 100 replicates.

As shown by the phylogenetic analysis of the nucleoprotein gene (Figure 7), the three vaccine strain Nishigahara, NI-CE and RC-HL were grouped with aG most closely.

## 4. Discussion

In the present study, the entire aG genomic sequence, which is composed of 11925 bp, was determined by 12 paris overlapping fragments covering the whole genome to improve our knowledge of the genetic relationship between aG strain and other strain of rabies virus available in Genbank. In contrast to the previous report [14], the length of the whole genomes in GT 1 lyssaviruses varies from 11711-nt to 12278-nt, with both even and odd numbers of nucleotides being represented [15,8]. In contrast to previous studies, the laboratory adapted vaccine strain aG has a odd nucleotide, while other laboratory isolates, such as Nishigahara, PV and SAD-B19 strain, have genomes of even nucleotides numbers [16]. It seems that genomes of odd numbers of nucleotides are more common in wild type strains than fixed strain but both odd and even numbers of genomes occurred in wild type strains and fixed strain. It remains to be determined that whether the isolates with odd or even numbers of the genome has any biological significance. However, the number of the sequence does not follow the 'rule of six' [17].

On average, the G+C content of the lyssaviruses genome is 44.57%, the G+C content was 45.18 mol% for aG strain. This is in agreement with the idea that the G+C content of the negative-stranded RNA viruses is lower than that of positive stranded RNA viruses, which has been considered to be associated with host cell RNA editing [18]. In agreement with previous studies, the genome of aG strain is similar to that of other lyssaviruses with few variations. The complete nucleotide length of aG strain is 11,925nt, which was one, seven and three nucleotides shorter than that of Nishigahara, PV and SAD-B19 strain. Transcriptional initiation and termination signals of aG strain at the beginning and end of the each monocistron, including the motif AACAYYHCT initiatite transcription and a nine nucleotide motif WG(A)_7_, except the AGAAAAAAA TTP at the N-P junction, which is involved in transcription termination and polyadenylation (TTP), are precisely the same as described by Marston. All the intergenic sequences (IGS) in the aG agree precisely with what was described in that report. Unlike PV and SAD strains, the G-L intergenic region of aG strain has only one polyadenylation site. The 3' and 5' of the genome not only play an important role in transcription, replication and the switching between these two functions, but also in the initiation of encapsidation. The 11 nts at the leader and trailer region of the genome were complementary conserved. These regions showed that the basic structure of rabies virus have been retained in strain aG [19].

It is reported that the 3'NCR of G mRNA to be the pseudogene, because there were two TTP motifs in PV. However, in aG strain, there is only one TTP motif, which holds true for many other isolates, including laboratory fixed strains and street rabies virus strains. Thus, a conclusion can be drawn that the psdudogene is only part of non-translated region of G mRNA.

It is assumed that the aG strain in China is more closely with rabies virus in northern and northeast part of China, since these strains are closely related geographically and ethnically. The phylogenetic tree based on the N gene showed that aG strain clustered with the Japanese vaccine strain, supporting the idea that rabies virus from the same region tend to cluster together.

Consistent with previous observations that viruses from the same geographical area tend to group together, the aG strain tends to cluster most closely with DRV, which was isolated from deer in Jilin province, China. Also, the aG strain grouped closely to other vaccine strain, especially the vaccine strain from Japan [20]. However, the aG strain did not share high nucleotide homology with wild strain, such as FJ008, FJ009 and HN10, making aG not the best vaccine in China [21], although the strain aG has been employed for human vaccine strain in China for a long time. Of course, more research is needed to confirm the hypothesis.

To date, several methods, such as rapid amplification of cDNA (RACE) [22-24] and gene-walking approach have been employed to obtain the whole genome sequence, including the 5' and 3' untranslated regions (UTR). In the present study, a simple method of amplification and sequencing of full length of rabies virus genome was performed, according to reference 7. Before this study, similar method has been used to sequence the genome of different viruses [25]. Compared with the method previously, the method described here is easy to perform, does not need complex principle and does not need expensive device; thus it is recommended for other virus.

Comparison of nucleotide sequences of rabies virus available in Genbank with aG strain showed extensive divergence excluding the transcription regulatory signals and limited stretches of the nucleotide. However, according to the phylogenetic analysis of the N gene of aG strain, the strain probably emerged from the DRV, or they may share the same ancestor, which was isolated deer in Jilin province, China. More researches are needed to determine whether the aG strain came originally as a result of the DRV adapting to the dog host or not. The Ser_389_, the putative casein-type phosphorylation site and regulation of viral RNA transcription and replication was conserved in strain aG. The antigenic sites in strain aG were conserved, indicating the antigenicity of aG was well retained, which was suitable for vaccine production.

P has been considered to be the most divergent of the five coding protein. The LC8-interacting motif was conserved in aG strain, indicating that the motif serve to transport the virus through neurons. Also, the L-binding region of P, as well as the N-binding site of P was also conserved, except Thr_174_, Glu_295_. These results, together with the five Ser and four Met, indicating that the same function which have been reported in fixed strains were also retained in aG strain. Mebatsion and Rasalingam questioned the function of the interaction between P and LC8 by constructing of recombinant virus in the absence of the LC8 binding site. The results indicated that the virus lacking the binding site was as pathogenic as wild virus, suggesting the interaction is not indispensible to the spread of RV.

M protein has been considered to be important in membrane budding and interacting with cellular components. Finke found that substitution of Arg_58 _to Gly_58 _reduced the expression of full-length RNA. The aG strain retains the Glu_58_, suggesting that it may serve to RNA regulation.

The G protein is related to cell attachment, responsible for the induction of neutralizing antibodies and cell-mediated immune response [26]. Ala_242_, Asp_255_, Ile_268_, and Arg_333_, which are correlated with pathogenicity of rabies virus, were encoded Cys, Met, Thr and Ala respectively. The antigenic sites were all retained in aG. These results suggest the antigenicity of G protein was retained. However, the strain aG was strongly attenuated, although other factors may be responsible for the pathogenicity of rabies virus.

The L protein, together with P compose RNA polymerase complex, which is involved in enzymic activities of polymerase. The RNA-binding region, active site of polymerase, the Pro residues rich motif GXGXG, which is involved in polyadenylation or protein kinase activities, were conserved; These motifs, together with GHP were conserved in strain aG. All These results suggest the L protein possess the same function as other strain. However, little is known about the mechanism of the function of L protein, therefore, more researches are needed and necessary.

## Conclusion

In this report, we analyzed the full genome of China human rabies vaccine strain aG. Our studies indicated that the genome of aG retained the basic characteristics of RV, including the organization of the genome, the functional domains of the each ORF. At gene level, N was the most conserved among the five coding genes, indicating We this gene is the most appropriate for quantitative genotype definition. The phylogenetic analysis of the N indicated the aG strain clustered most closely with Japanese and Russian rabies vaccine strains, suggesting that they may share the same ancestor; also, the aG strain did not share high homology with wild strains isolated from China, making it may not be the best vaccine strain, more research is needed to elucidate the genetic relationship among the RV circulating in China.

## List of abbreviations

RACE: rapid amplification of cDNA ends; RV: rabies virus; RT-PCR: Reverse transcription polymerase chain reaction; RNP: ribonucleoprotein; PHKC: primary hamster kidney cell.

## Competing interests

The author declares that they have no competing interests.

## Authors' contributions

WQJ, XPY carried out the molecular genetic studies, participated in the sequence alignment and drafted the manuscript. ZYL, XL, XRL, XTT, BYL, BY participated in the design of the study and helped to draft the manuscript. JXL* is the corresponding author. All authors have read and approved the final manuscript.

## Author Details

State Key Laboratory of Veterinary Etiological biology, Key Laboratory of Epizootic disease of Grazing Animal of Ministry of Agriculture, Lanzhou Veterinary Research Institute, Chinese Academy of Agricultural Science (LVRI, CAAS), Lan Zhou, 730046, China.
